# Vimentin regulates Notch signaling strength and arterial remodeling in response to hemodynamic stress

**DOI:** 10.1038/s41598-019-48218-w

**Published:** 2019-08-27

**Authors:** Nicole C. A. van Engeland, Freddy Suarez Rodriguez, Adolfo Rivero-Müller, Tommaso Ristori, Camille L. Duran, Oscar M. J. A. Stassen, Daniel Antfolk, Rob C. H. Driessen, Saku Ruohonen, Suvi T. Ruohonen, Salla Nuutinen, Eriika Savontaus, Sandra Loerakker, Kayla J. Bayless, Marika Sjöqvist, Carlijn V. C. Bouten, John E. Eriksson, Cecilia M. Sahlgren

**Affiliations:** 1 Åbo Akademi University, Faculty of Science and Engineering, Biosciences, Turku, Finland; 2 Eindhoven University of Technology, Department of Biomedical Engineering, 5600 MB Eindhoven, The Netherlands; 3 Turku Bioscience, Åbo Akademi University and University of Turku, Turku, Finland; 4 Department of Biochemistry and Molecular Biology, Medical University of Lublin, Lublin, Poland; 5 Department of Molecular & Cellular Medicine, Texas A&M University Health Science Center, College Station, TX 77843 Texas, USA; 6 Institute of Biomedicine, Research Centre for Integrative Physiology and Pharmacology, University of Turku, Turku, Finland; 7 Institute of Complex Molecular Systems, Eindhoven University of Technology, Eindhoven, The Netherlands; 8 Turku Center for Disease Modelling, University of Turku, Turku, Finland

**Keywords:** Cell signalling, Intermediate filaments, Cellular microbiology

## Abstract

The intermediate filament (IF) cytoskeleton has been proposed to regulate morphogenic processes by integrating the cell fate signaling machinery with mechanical cues. Signaling between endothelial cells (ECs) and vascular smooth muscle cells (VSMCs) through the Notch pathway regulates arterial remodeling in response to changes in blood flow. Here we show that the IF-protein vimentin regulates Notch signaling strength and arterial remodeling in response to hemodynamic forces. Vimentin is important for Notch transactivation by ECs and vimentin knockout mice (VimKO) display disrupted VSMC differentiation and adverse remodeling in aortic explants and *in vivo*. Shear stress increases Jagged1 levels and Notch activation in a vimentin-dependent manner. Shear stress induces phosphorylation of vimentin at serine 38 and phosphorylated vimentin interacts with Jagged1 and increases Notch activation potential. Reduced Jagged1-Notch transactivation strength disrupts lateral signal induction through the arterial wall leading to adverse remodeling. Taken together we demonstrate that vimentin forms a central part of a mechanochemical transduction pathway that regulates multilayer communication and structural homeostasis of the arterial wall.

## Introduction

The vascular wall is subjected to physical forces from blood flow, and arteries form and adapt their structure to maintain mechanical homeostasis in the hemodynamic environment^1–4^. In response to changes in blood flow, endothelial cells (ECs) lining the vessel lumen respond and communicate with vascular smooth muscle cells (VSMCs) to control structural remodeling^5–9^. In a homeostatic healthy vessel, VSMCs exhibit a contractile differentiated phenotype. Long term changes in e.g. blood pressure lead to vascular remodeling, where VSMCs switch from a contractile differentiated phenotype to a proliferative one.

The intermediate filament (IF) proteins are critical mechanical components of the cell and maintain cell and tissue integrity^10^. ECs and VSMCs express the IF protein vimentin, and high content of vimentin is found in larger arteries such as the aorta and the carotid artery. The vimentin network is important for vascular cell and tissue integrity during hemodynamic stress^11–14^ and plays a role in arterial compliance^15^. Vimentin also regulates actin stress fiber assembly and contractility^16–18^. Vimentin acts as a scaffold for signaling proteins that regulate epithelial mesenchymal transition (EMT), cancer cell invasion, wound healing and tissue morphogenesis, angiogenesis, as well as inflammatory signaling in fibrosis and graft-versus-host disease^19–25^. The interaction with signaling proteins is regulated by post translational modifications. In VSMCs phosphorylation of vimentin at serine 55/56 plays an important role in regulating smooth muscle cell force transmission and contraction^26–31^. How vimentin integrates mechanics with EC-VSMC signaling is not known.

Notch signaling regulates the development and structure of the arterial wall^32–37^. The Notch ligand Jagged1 plays an especially important role in patterning of the vascular wall. EC-specific knockout of Jagged1 is embryonic lethal due to defective VSMC differentiation^38,39^, and reduced levels of endothelial Jagged1 leads to loss of VSMCs and vascular regression^40^. Aging leads to impaired Jagged1 expression in ECs after vascular injury and enhances VSMC proliferation^40^. Jagged1 in VSMCs relays the signal for differentiation deeper into the artery wall, through lateral induction, where Notch activation leads to expression of Jagged1 in the signal receiving cells to activate Notch in the next cell layer^35,41–43^. Continuous Jagged1 signaling in VSMCs is important to maintain VSMC phenotype and the arterial structure^44^ and Jagged1 knockout in VSMCs results in loss of differentiated VSMCs^34,35,41^. We have previously demonstrated that vimentin interacts with the Notch ligand Jagged1^21^, but how the interaction is regulated and whether vimentin plays role in EC-VSMC signaling through Jagged1 is not known.

## Results

### Vimentin regulates VSMC coverage through Jagged1

**Figure 1:**
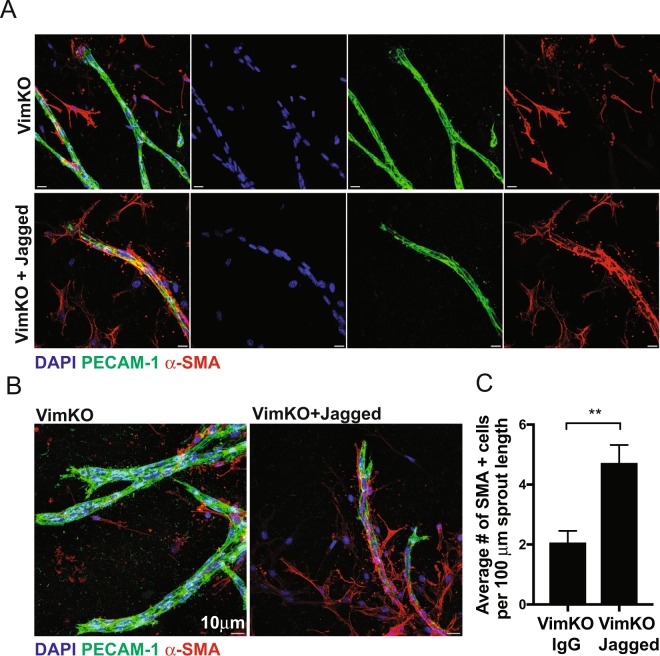
Vimentin regulates VSMC coverage and differentiation in aortic rings through Jagged1. Aortic ring assays were performed using aortae from VimWT and VimKO mice. Recombinant IgG-Fc or Jagged1-Fc proteins were conjugated to Protein A Agarose beads and added to collagen matrices. After 7 days, rings were fixed, permeabilized, and stained with DAPI (blue) and antibodies directed to PECAM-1 (green) and alpha-smooth muscle actin (αSMA, red). Using confocal microscopy, Z-stack images were captured with a 1 µm step size. Representative images as max projections are shown. Scale bar represents 10 µm. Using Z-stacked images captured in (**A**,**B**), the number of αSMA positive cells along the length of the PECAM-1 positive structure was quantified (**C**). Data represent the average number of αSMA-positive cells per 100 µm EC sprout length. Error bars represent SEM. Statistical significance was determined using a Student’s t-test, p < 0.01.

We used an *ex vivo* aortic ring assay to assess the importance of vimentin in regulating EC-VSMC signaling and VSMC coverage. Aortae from vimentin wildtype (VimWT) and vimentin knock-out (VimKO) mice were harvested, cut into rings, embedded into collagen, and fed medium supplemented with VEGF to induce endothelial sprouting^21^. Endothelial sprouts and differentiated smooth muscle cells were identified through immunostaining for PECAM-1 and αSMA, respectively (Supplementary Fig. 1). In line with our previous observations, loss of vimentin decreased endothelial sprouting responses, compared to VimWT controls^21^. Quantification of the number of VSMCs expressing αSMA along the endothelial outgrowths revealed that VimKO endothelial sprouts exhibited significantly reduced smooth muscle cell coverage, compared to the VimWT (Supplementary Fig. 1). We next tested if the VSMC dysregulation was due to derailed Jagged1 signaling and whether Jagged1 reactivation could rescue the VSMC phenotype in VimKO aortic rings. To this end, we immobilized recombinant Jagged1 carrying an IgG domain to IgG-binding beads in the collagen matrix to obtain the resistance needed for Notch activation. Activation of Notch signaling using immobilized Jagged1 significantly enhanced the αSMA positive cell coverage from VimKO aortic rings, while IgG controls had no effect (Fig. 1A,B).

### Vimentin knock out mice display adverse arterial remodeling

**Figure 2:**
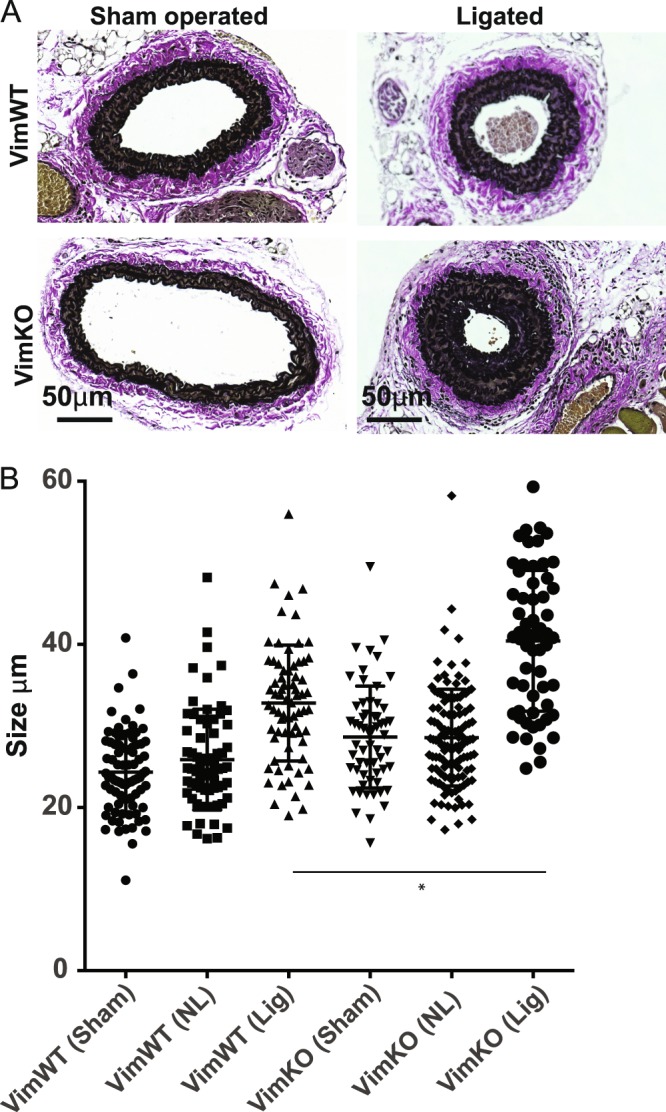
VimKO mice demonstrate adverse remodeling responses after carotid ligation. Arterial remodeling in VimWT and VimKO mice was analysed 4 weeks after ligation of the left carotid artery. (**A**) Staining of collagen and elastin in sham operated (left image) and ligated (right image). VimKO mice display increased thickening of the arterial wall compared to VimWT mice. (**B**) The thickness of the VSMC layer in VimWT and VimKO carotids in sham operated and operated mice was measured. (Sham) sham-operated; (NL) contralateral non-ligated; (Lig) ipsilateral ligated. Statistical analysis confirm that the vessel wall was significantly thicker in the ligated VimKO artery compared to VimWT. ANOVA was used for statistical analyses, followed by Tukey-Kramer multiple comparisons post hoc test to identify the groups differing. Data is presented as the mean ± SD, and p < 0.05 was considered statistically significant.

**Figure 3:**
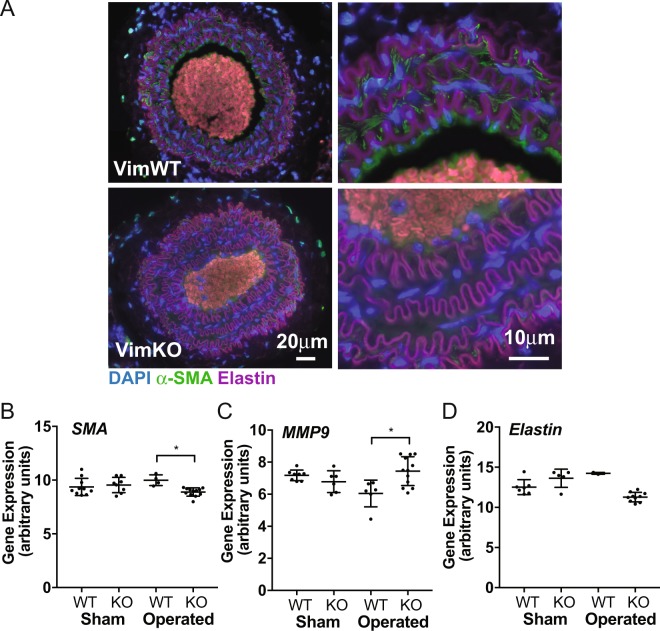
VimKO mice demonstrate changed expression of VSMC phenotypic markers in remodeling arteries. Arterial remodeling and expression of VSMC phenotype specific markers in VimWT and VimKO mice were analysed by immunostaining and Q-PCR of isolated VimWT and VimKO carotid arteries after ligation. (**A**) Smooth muscle actin (αSMA) staining is reduced in VimKO carotid arteries 4 weeks after carotid ligation. **(B**–**D)** Analyses of expression of *αSMA, MMP9* and *elastin* in VimWT and VimKO mice. Expression of* αSMA* and *elastin* was reduced and expression of *MMP9* was increased in the contralateral VimKO artery 4 weeks after ligation. ANOVA was used for statistical analyses, followed by Tukey-Kramer multiple comparisons post hoc test to identify the differing groups. Data is presented as the mean ± SD, and p < 0.05 was considered statistically significant.

To assess if vimentin plays a role in arterial remodeling *in vivo*, we ligated the left carotid artery of VimWT and VimKO mice, and analysed arterial structure, and expression of VSMC markers in the contralateral artery after 4 weeks. The carotid artery in non-operated VimKO mice were slightly stiffer and displayed abnormal contraction and relaxation responses (Supplementary Fig. 2), in line with recent observations^15^. In response to the ligation, the ligated artery remodeled and the radius of the arterial wall increased in both groups (Fig. 2A,B). The arterial wall in VimKO mice was significantly thicker than the VimWT wall at 4 weeks after the ligation (average 40 μm for VimKO, as compared to 30 μm for VimWT) (Fig. 2A,B). The VimKO artery displayed reduced αSMA staining and *αSMA* expression (Fig. 3A,B) and increased expression of *MMP9*, a marker for proliferative VSMCs, (Fig. 3C), indicating that differentiation of VSMCs was hampered. The expression of elastin, which maintains the differentiated VSMC phenotype, was also downregulated in the VimKO (Fig. 3D). In sum, the data show that the VimKO arteries display adverse remodeling with a more persistent switch of VSMCs to a synthetic non-differentiated phenotype.

Vimentin deletion affected the expression of Notch ligands, receptor and target genes. Expression of *Notch3*, *Hes1*, *Hey1 and Dll1* were significantly increased in the contralateral artery 4 weeks after occlusion in VimKO mice as compared to VimWT mice (Supplementary Fig. 3). Expression of *Hey2* was significantly reduced (Supplementary Fig. 3) and there was no change in *Jagged1* expression (Supplementary Fig. 3). We also observed an increase in the expression of *Dll4* in both VimWT and VimKO arteries with no significant difference between the groups (Supplementary Fig. 3). The data indicate that loss of vimentin leads to a disrupted Notch signaling profile during arterial remodeling in response to hemodynamic stress *in vivo*.

### Shear stress induced vimentin phosphorylation increases Notch activation

**Figure 4:**
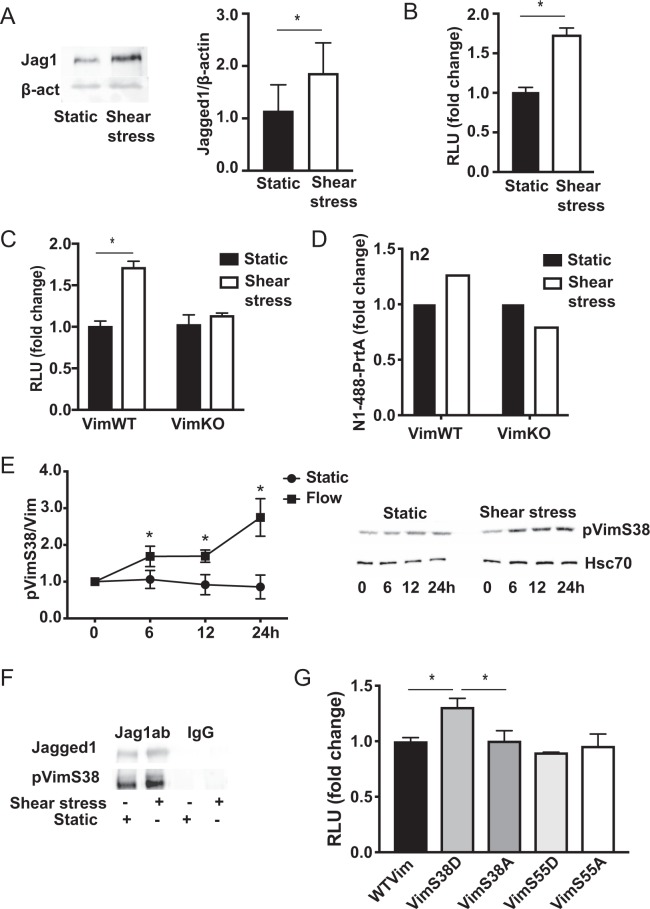
Shear stress induces vimentin phosphorylation and Jagged1 interaction and enhances Jagged1-Notch transactivation. (**A**) Jagged1 protein expression in ECs cultured under static conditions or under shear stress analysed by western blotting. The graph shows quantification of Jagged1 levels in three independent experiments. (**B**) Shear stress enhances the signal sending ability of ECs, as demonstrated by increased Notch activity in reporter cells co-cultured with ECs exposed to either static or shear stress conditions. (**C**) Shear stress enhances the signal sending ability of vimentin expressing cells (VimWT) but not vimentin depleted cells (VimKO). (**D**) Shear stress enhances N1ECD-Jagged1 endocytosis in VimWT but not in VimKO cells. Fluorescently labelled N1ECD was coupled to Protein A (PrtA) beads (N1-488-PrtA) in order to mimic the mechanical strain produced during receptor-ligand endocytosis and transactivation and N1-488-PrtA uptake was analysed by FACS. The graph shows data from 2 separate experiments. (**E**) Shear stress induces vimentin phosphorylation. Expression levels of vimentin phosphorylated at serine 38 in ECs exposed shear stress as analysed by western blotting using phosphospecific antibodies. (**F**) Jagged1 interacts with phosphorylated vimentin. Jagged1 was immunoprecipitated from ECs cells under static and shear stress conditions and the interaction with phosphorylated vimentin was assessed by immunoblotting of the precipitate by phosphospecific antibodies. **(G**) Vimentin phosphorylation at serine 38 enhances Notch activation potential. Jagged1 expressing and vimentin depleted cells were transfected with wildtype vimentin (WTVim), phosphomimicking forms of vimentin (Vim38D or Vim55D) or phospho-dead forms of vimentin (Vim38A or Vim55A). The Notch activation potential of the cells was measured by coculturing the cells with Notch reporter cells. The graph shows data from four separate experiments.

We have previously shown that vimentin interacts with Jagged1 to promote robust signal activation^21,45,46^. The interaction was verified by proximity ligation assay, and immunoprecipitation analyses indicated that shear stress enforced the vimentin Jagged1 interaction (Supplementary Fig. 4A,B). Shear stress further increased Jagged1 levels (Fig. 4A) and enhanced Notch signal activation as measured by reporter cells (Fig. 4B). To assess if vimentin is important for efficient Notch transactivation during shear stress we co-cultured the Notch reporter cells with VimWT and VimKO cells exposed to shear stress. Shear stress enhanced Notch activation by VimWT cells but not by VimKO cells (Fig. 4C). Ligand trafficking is important in Notch signaling and ligand endocytosis after receptor binding is required for efficient receptor activation^46,47^. To analyse ligand endocytosis during shear stress we incubated VimWT and VimKO cells expressing Jagged1 with a fluorescently labelled peptide mimicking the Notch extracellular domain linked to beads (N1-488-PrtA) to resemble the mechanical strain raised during receptor transactivation. The cells were exposed to shear stress and internalization of N1-488-PrtA was measured by FACS. Shear stress enhanced internalization of N1-488-PrtA in VimWT cells but not in VimKO cells (Fig. 4D). The data demonstrate that vimentin is important for Jagged1 mediated Notch transactivation during shear stress.

Association of vimentin with signaling proteins has been shown to be regulated by vimentin phosphorylation and vimentin assembly state^48,49^. Shear stress did not significantly affect vimentin assembly state (Supplementary Fig. 4C)^50^, but induced displacement of the network away from the cell membrane (Supplementary Fig. 4D). To assess vimentin phosphorylation during shear stress we used a panel of phospho-specific vimentin antibodies. Shear stress increased vimentin phosphorylation at serine 38 (Fig. 4E). Immunoprecipitation analyses demonstrated that Jagged1 interacted with vimentin phosphorylated at serine 38. In contrast to phosphorylation at serine 38, phosphorylation at serine 55 did not increase upon shear stress although shear stress did induced co-localization of Jagged1 and serine 55 phosphorylated vimentin in cytoplasmic clusters (Supplementary Fig. 4E,F). To assess the importance of vimentin phosphorylation for Notch transactivation we co-cultured cells expressing wildtype vimentin and phospho-mimicking (serine to aspartic acid substitution: Vim38D, Vim55D) and phospho-dead (serine to alanine substitutions: Vim38A, Vim55A) variants of vimentin with Notch reporter cells (Fig. 4G). Phosphorylation of vimentin at serine 38, but not at serine 55 increased Notch transactivation potential. Taken together the data indicate that shear stress induced site specific vimentin phosphorylation that regulates Jagged1 interaction and Notch transactivation.

### Strain induces vimentin assembly and reduces Notch activity

Whereas ECs are exposed to shear stress, VSMCs mainly experience strain, high strain in thinner vessels and low strain in thicker vessels. Jagged1-Notch signaling is propagated in the VSMC layers in the arterial wall through lateral induction to regulate the phenotype of VSMCs and re-establish homeostasis in response to biomechanical input. To analyse the effect of strain on vimentin and Notch signaling, we exposed VSMCs to 10% uniaxial strain. Straining decreased the amount of vimentin in the soluble fraction and increased the amount of vimentin in the insoluble fraction (Supplementary Fig. 5A), demonstrating that shear stress and strain have distinct effects on vimentin, and strain induces vimentin filament polymerization. Whereas vimentin shifts to the insoluble pool, the abundance of Jagged1 is found in the soluble pool (Supplementary Fig. 5B). Immunoprecipitation of filamentous vimentin and quantitative co-localization studies of abundant vimentin filaments with low abundance signaling molecules using immunocytochemistry is challenging but the fractionation data suggest that Jagged1 separates from vimentin upon increasing strain. Q-PCR analyses of strained VSMC demonstrated reduced expression of *Notch3*, *Jagged1* and Notch target genes *Hey1*, *Hey2* and *Hes1* (Supplementary Fig. 5C). The data are in line with ours and others previous observations, and indicates that Notch activity decreases with increasing strain.

### Reduced Jagged1-Notch transactivation in the absence of vimentin explains adverse arterial remodeling *in vivo*

**Figure 5:**
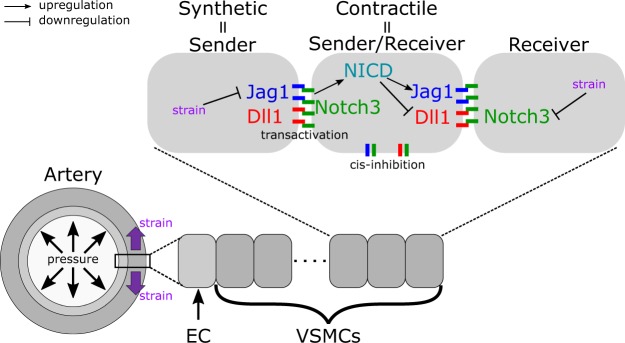
A schematic representation of the computational model of Notch signaling in the arterial wall. For more information please see supplementary material and methods.

The multifaceted effects of vimentin depletion *in vivo* challenge the identification of the underlining mechanism for the adverse remodeling in the VimKO artery. We used a computational model of Notch signaling in the arterial wall^51^ to investigate if the loss of VSMC differentiation and the thickening of the arteries in the VimKO mice could be explained by reduced transactivation in the absence of vimentin. The model is able to predict the homeostatic thickness of muscular arteries at different locations of the arterial tree, see supplementary materials and methods for details. In the model (Fig. 5), VSMCs express Dll1, Jagged1, and Notch3 and downregulate Jagged1 and Notch3 production in response to strain, as previously experimentally verified and supported by the data in the present work (ref.^51^, Supplementary Fig. 5). The VSMC phenotype is computed based on the level of Notch3 intracellular domain (NICD) translocated to the nucleus due to Jagged1- or Dll1-induced transactivation (proliferative for NICD < 100 molecules and contractile for 100 < NICD < 300). The simulations predict that VSMCs collectively switch phenotype, from proliferative for relatively thin vessels (high strain) to contractile for relatively thick vessels (low strain). In healthy VimWT conditions, the wall thickness threshold of the phenotypic switch corresponded to the homeostatic wall thickness.

**Figure 6:**
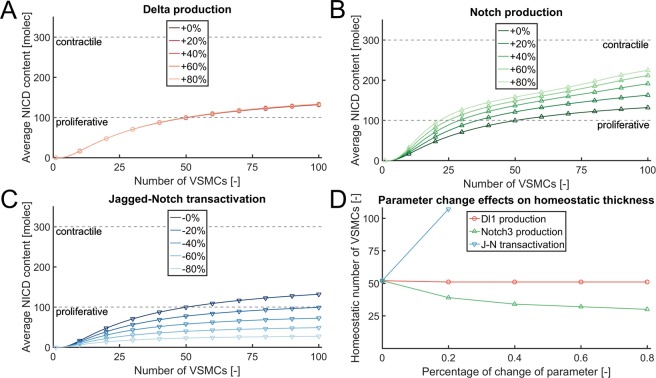
Loss of Jagged1-Notch3 transactivation strength explains the thickening of VimKO arteries. Results of the computational simulation of Notch signaling through arterial layers with parameter variations to investigate the effects of VimKO. The original model parameters are reported in the supplementary material. Average NICD content across the layers, with parameter variations. (**A**) Changes of Dll1 production have little effects on the average NICD content. (**B**) Increases of Notch3 production induce higher average NICD contents. (**C**) Decreases of Jagged-Notch transactivation strength correspond to lower average NICD contents. (**D**) Similar trends can be observed with respect to the homeostatic number of VSMC layers, computed as the arterial thickness for which the model predicts a switch-type transition from proliferative to contractile VSMC phenotype.

We first tested whether increasing or decreasing Jagged1 production affected arterial thickness. An increase in Jagged1 production did not have a significant effect. However, a decrease in Jagged1 production by 50% and 75% led to a switch of VSMCs to a proliferative phenotype (Supplementary Fig. 6), demonstrating that Jagged1 signaling is required to maintain the differentiate VSMC phenotype and structural homeostasis. We next performed simulations decreasing the computational parameter associated with Jagged1-Notch transactivation, the component in the signaling process directly affected by vimentin according to our *in vitro* analyses (Fig. 4). In the computational results, this decrease corresponded to a decrease of the average NICD values (Fig. 6C), with a consequent increase of the homeostatic thickness predicted by the simulations (Fig. 6D) and demonstrated by the *in vivo* experiments (Fig. 2). We then investigated whether the individual increases in Dll1 and Notch3 expressions observed in VimKO arteries (Supplementary Fig. 3) could cause the thickening of these arteries, by increasing the computational parameters associated with their production rates. Variations of Dll1 production (Fig. 6A) had no significant effects on the average NICD content per layer, and thus on the homeostatic arterial thickness (Fig. 6D). An increased Notch3 production in the computational model corresponded to higher average values of NICD content (Fig. 6B), and therefore to thinner arteries predicted by the simulations (Fig. 6D). Taken together the data suggest that a reduced Jagged-Notch transactivation strength leads to a switch to a proliferative VSMC phenotype and the thickening of VimKO arteries. This notion was further supported by the observation that Jagged1, but not Dll1, regulated the expression of the VSMC differentiation marker αSMA (Supplementary Fig. 7).

## Discussion

The cytoskeletal network of the cell responds to mechanical cues from blood flow^52,53^ and intermediate filament proteins have been suggested to play a role in adaptive behavior in response to changes in blood flow^54–56^. Here we show that vimentin is important for re-establishing structural homeostasis of the carotid artery in response to hemodynamic stress. Vimentin regulates VSMC phenotype and arterial remodeling through Notch signaling. VimKO mice display disrupted VSMCs and adverse structural remodeling of the artery in response to hemodynamic stress induced by experimental carotid occlusion. A recent publication implicated vimentin in the regulation of basement membrane composition and tissue stiffness^15^. In line with their report, we observed changes in the mechanical properties of the artery and disrupted responses to contractile and relaxation stimuli (Supplementary Fig. 2). Further, vimentin has been shown to regulate the dynamics of actin thereby affecting an important force generator of cells^18^.

We demonstrate that vimentin forms mechanoresponsive structures and tunes Notch signaling strength in response to hemodynamic forces. Notch signaling is strictly dose sensitive and both hyper- and hypo-activity is linked to cardiovascular disease^57–59^. The prevalence of diseases related to Notch in mechanically loaded tissue imply a strong link between mechanical cues and pathological changes in Notch signaling. These mechanical links include loss of anti-calcifying responses under flow in Notch1-depleted ECs^60^, contraction-induced Notch-dependent formation of trabeculae in the developing zebrafish heart^61^, flow induced reduction of Notch expression during lymph-vessel sprouting^62^, Notch3 dependent contractility of cerebral arteries in CADASIL^63^, blood flow dependent angiogenesis of skeletal vasculature^64^ and vascular barrier function^65^. In several of these cases vimentin may play a role, warranting further examination of the involvement of vimentin in mechanically linked cardiovascular pathogenesis.

Mechanistically, vimentin interacts with Jagged1 and regulates Jagged1-Notch transactivation. Our data show that hemodynamic forces regulate the interaction between vimentin and Jagged1 in a vimentin phosphorylation- and assembly-state specific manner. Shear stress induces vimentin phosphorylation at serine 38 and interaction with Jagged to enhance Jagged1-Notch transactivation. Although shear stress induced co-localization of vimentin phosphorylated at serine 55 and Jagged1 in subcellular clusters, serine 55 phosphorylation did not increase in ECs in response to shear stress and did not enhance Notch transactivation. Vimentin is phosphorylated at serine 55 in VSMCs and phosphorylation is linked to VSMC force transmission and contraction^26–30,49^. Given the role of vimentin in vesicle trafficking and the importance of Notch ligand endocytosis, one could speculate that site specific phosphorylation of vimentin co-ordinates Notch ligand endocytosis to tune signaling strength. In line with this notion, PKCε-mediated phosphorylation of vimentin has been demonstrated to regulate integrin trafficking and cell motility^24^. Further efforts should be placed on elucidating the role of vimentin modifications in trafficking check point control.

Based on our data, we propose that vimentin is an integral part of Notch signaling in the vasculature and that it regulates Notch signaling strength in response to changes in hemodynamic conditions. Mechanical tuning of Jagged1 is enabled due to its catch-bond behavior^45^. Ligand-mediated transendocytosis of the receptor produces a strain on the receptor, a process that requires assembly of actin filaments at the membrane^46^. Recent data demonstrate that actomyosin contractility regulates Notch signaling *in vivo*^66^. Myosin II dependent tension is important in signaling both in the signal sending and the signal receiving cell, but contributes most to signaling on a distance through cellular protrusions although also required for robust signaling between adjacent cells, demonstrating that cytoskeletal forces contributes to signaling. Interestingly, actomyosin contractility and ligand endocytosis influence Notch signaling *in vivo* in distinct ways via protrusion- and lateral cell-cell contact-mediated signaling, respectively. Both systems are important for robust Notch activation. Mammalian cells express IFs that also influence Notch signaling^67–69^. The tissue- and developmental-stage specific expression of IF proteins, the interaction between different IF systems in a cell, and the dynamic and flexible regulation of IFs through posttranslational modifications induced by input from the environment offer an excellent way for fine tuning signalling strength and cellular responses. Further, a bidirectional crosstalk exists between vimentin and the actin cytoskeleton^16,18^. We show that site-specific modifications of vimentin induced by mechanical stresses regulate Notch signaling strength. Further efforts should be placed on identifying factors that regulate vimentin and on elucidating how these affect the vimentin-Jagged1-Notch axis in the vasculature. Importantly, specific regulation of different components of the Notch pathway in response to mechanical stress will contribute to Notch signaling strength^16,51,65,70–72^. VSMCs also express the intermediate filament protein desmin, a vimentin assembly partner that plays an important role in VSMC and muscular biology^54,73^. The current study does not consider the role of desmin in the VimKO phenotype, although an interplay between desmin and vimentin could affect mechanosensing and signaling. Importantly, overexpression of Jagged1 has been shown to rescue the Duchenne Muscular Dystrophy Phenotype, caused by a mutation in the dystrophin gene^74^. Dystrophin links the cytoskeleton to the extracellular matrix and has been implicated in adaptive responses to chronic changes in blood flow^75,76^. A potential link between dystrophin, desmin, vimentin, and Jagged1 should be given further consideration.

In sum, we have demonstrated that the intermediate filament protein vimentin regulates Notch signaling strength and structural remodeling of the artery. Given our findings, the possibility of targeting vimentin and other mechanosensitive regulators of Notch signaling warrants further investigation as it can be expected to lead to new therapies.

## Materials and Methods

### Human umbilical vein endothelial cells

HUVECs were cultured in endothelial cell media 2 (Promocell) supplemented with Fetal Calf serum (0.02 ml/ml), Epidermal growth factor (5 ng/ml), Basic fibroblast growth factor (10 ng/ml), Insulin-like growth factor (20 ng/ml), Vascular endothelial growth factor (0.5 ng/ml), Ascorbic acid (1 µg/ml), Heparin (22.5 µg/ml) and hydrocortisone (0.2 µg/ml). Cells were seeded and expanded on tissue culture polystyrene (TCPS) at 37 °C, 5% CO_2_ and medium was changed every 3–4 days.

### Human aortic vascular smooth muscle cells

HAoSMCs were cultured in Smooth Muscle Growth Medium-2 (Lonza, SMGM-2), supplemented with the SMGM-2 SingleQuot Kit Suppl. And Growth Factors (Lonza) and 1% v/v penicillin/streptomycin (P/S, Sigma Aldrich). Cells were seeded and expanded on tissue culture polystyrene (TCPS) at 37 °C, 5% CO_2_ and medium was changed every 3–4 days.

### Mouse embryonic fibroblasts

MEFs were cultured in Advanced Dulbecco’s Modified Eagle Medium (DMEM, Gibco) supplemented with 1% v/v P/S, 2mM L-Glutamine (Sigma), and 5% fetal bovine serum. Cells were seeded and expanded on tissue culture polystyrene (TCPS) at 37 °C, 5% CO_2_ and medium was changed every 2–3 days.

### Animals

The mice were kept in specific pathogen-free conditions, 2–4 per cage, in controlled conditions of light (12 h light, 12 h dark) and temperature (21 ± 1 °C). The mice were fed with mouse chow CRM3 (soy free, Special Diet Service) and tap water ad libitum. Animals were cared for in accordance with the Directives 2012/707/EU, 2014/11/EU and of the European Parliament and of the Council for the Care and Use of Laboratory Animals. All animal experimental protocols were approved by the Board of Animal Experiments at the Regional State Administrative Agency of Finland (ESAVI/438/04.10.03/2012, ESAVI/1256/04.10.07/2015, ESAVI/8305/04.10.07/2013). In all experiments WT and heterozygous littermates were used as controls.

### Aortic rings

Aortae were harvested from eight-week-old VimWT and VimKO Sv129/Pas mice. Aortic rings were embedded into 1.5 mg/ml collagen type I and, after collagen polymerization, fed with Opti-MEM containing 2.5% FBS and 40 ng/ml VEGF. Aortic ring assays were incubated at 37 °C with 5% CO_2_, and after four days, the media was replaced.

### Aortic ring assay

Aortic ring assays were performed as previously described^21,77^. After six total days of incubation, aortic ring assays were fixed with 4% paraformaldehyde in PBS for twenty minutes and rinsed twice with Tris-Glycine (see supplemental materials for culturing protocols). Incubation of aortic rings with recombinant proteins attached to beads was performed as previously described^21^. A total of 1 μg of Recombinant Jagged1 Fc (R&D Systems, 1277-JG-050), or IgG Fc control (R&D Systems, 110-HG-100) were coupled to 10 μl Protein A agarose beads (Roche, 11719408001), diluted in PBS to give a final volume of 100 μl. The bead–ligand mixtures were gently rotated for 30 minutes at room temperature (RT), and the entire 100 μl mixture was added to 900 μl collagen type I, on ice, for a final collagen concentration of 2 mg/ml. Aortae were harvested as described above. A total of 70 μl of the collagen–bead mixture was added per well of a full area 96-well plate, three wells at a time, and 0.5-mm aortic rings were oriented so the lumen was visible. After the collagen polymerized, aortic rings were fed with 150 μl Opti-MEM containing 2.5% FBS and 40 ng/ml VEGF and incubated at 37 °C with 5% CO_2_. After four days of incubation, the media were replaced. Rings were fixed after six days with 4% paraformaldehyde in PBS and rinsed twice with Tris-Glycine.

### Immunostaining of aortic rings

Aortic rings were permeabilized for 30 minutes with 0.5% Triton X- 100 in PBS, then blocked overnight at 4 °C with blocking buffer (0.1% Triton X-100, 1% BSA, and 1% goat serum in TBS). Rings were immunostained using primary antibodies directed against PECAM-1 (Santa Cruz, sc-1505R), followed by incubation with Alexa-488 conjugated secondary antibodies (Invitrogen, A11008) and Cy3-conjugated alpha smooth muscle actin (αSMA, Sigma, C6198). Cell nuclei were stained with 1 μM DAPI (Invitrogen, D1306).

### Quantification of SMC coverage of EC sprouts

αSMA positive cell coverage of EC sprouts was quantified using Z-stacked confocal images of PECAM-1 and αSMA immunostained aortic rings. Z-stacks of images with 1 μm step-size of aortic rings were taken using a Nikon TI A1R inverted confocal microscope at 40× magnification. Images were compressed and analysed using Nikon Elements Software. A line was drawn along the EC sprout to measure the length, and the number of αSMA positive cells along the EC sprout was quantified. The average number of αSMA positive cells per 100 μm of vessel length was quantified from at least 13 sprouts per genotype. Error bars represent standard error of the mean (SEM).

### Detergent extraction and fractionation

Cells were washed, incubated with Triton Lysis Buffer for 15 minutes and collected. After centrifuging 15 min at 10.000 g pellet and supernatant were separated. Samples were analysed by SDS-page and westernblotting using antibodies recognizing vimentin (D21H3) (rabbit, Cell signaling) and Hsc70 (rabbit, Cell signaling).

### Receptor binding and ligand trans-endocytosis during shear stress

Mouse embryonic fibroblasts (MEFs) were plated on 12-well plates and transfected with Jagged1 using Xfect (Clontech) according to the manufacturer’s instructions (see supplemental materials for cell culture protocols). Recombinant Notch1-extracellular domain Fc Chimera rN1ECD (R&D Systems, Cat. No. 1057-TK) (final concentration 1 µg/ml) and Alexa Fluor 488 goat anti-human IgG (Invitrogen, Cat. No. A11013) (final dilution 1:2000) were diluted in sterile PBS and incubated on rotation in +4 °C for 30 min. The complex was further coupled to protein A agarose beads by incubation in +4 °C for 30 min. The rN1ECD-Alexa 488 solution diluted in cold DMEM was added to the cells, followed by incubation on ice for 30 min. The media was changed to warm and the cells were subjected to shear stress in the form of rotation (150 rpm) for 30 min in 37 °C. The cells were detached, centrifuged (450 g, 5 min) and fixed in 2% PFA for 5 min in RT, followed by resuspension in PBS and analysis by FACS.

### Shear stress experiments

For shear stress experiments with imaging as readout, HUVEC were seeded into collagen IV coated 6-channel slides from ibidi (μ-slide VI 0.4, #80606) at 10^6^ cells/ml. The next day HUVEC were exposed to 1 Pa shear stress for 24 hours or kept as a static control. Afterwards the cells were washed twice with PBS and fixed in 4% PFA for 20 minutes. Fixed cells were permeabilized with 0.1% Triton X-100 in PBS for 15 minutes and blocked with 4% donkey serum in PBS for 30 minutes. The cells were stained for Jagged1 (H-66, Santa Cruz) and S55 phospho-vimentin (ab22651, Abcam) overnight at 4 °C. Secondary antibodies (A21206 and A21146, ThermoFisher) were incubated for 45 minutes at RT. The nuclei were stained with DAPI (Sigma) for 5 minutes. After a final washing step with PBS Mowiol was injected into the channels as a mounting medium. The cells were imaged with a Zeiss Axiovert 200 M, using a 40x/0.95 objective.

### Notch reporter assay

Vim WT and KO cells were exposed to flow for 24 h. 12xCSL-luciferase transfected HEK293T cells were seeded directly on top and co-cultured for 24 hours. Afterwards the cells were lysed and luciferase reporter activity was measured with a Biotek Synergy plate reader using a luciferase Assay (Promega).

### Co-localization analysis

For 3D Jagged1 – S55 phospho-vimentin colocalization analysis we imaged the cells on a Leica TCS SP5X system. The cells were imaged with a 20x/0.7 objective, 1028×1028 frame size with 87 nm pixel size and 8 line averages. The cells were sequentially scanned for the green (488 nm excitation, 494–610 nm HyD) and red channel (555 nm excitation, 562–676 nm HyD). All confocal dataset were deconvolved using the Huygens Software (Scientific Volume Imaging, Hilversum). The microscopic parameters were set to a refractive index of 1.490 (for Mowiol) as embedding medium. For deconvolution we used a theoretical PSF, automated background estimation and the CMLE deconvolution algorithm. Afterwards the Spearman colocalization coefficient was calculated using Costes method for background estimation.

### Ligation left carotid artery

Mice were operated at 3-months of age by ligating the left carotid artery. Sham operated mice and non-operated mice were used as controls. A few days before and three days after operation the pressure gradient was measured using a Doppler echocardiography (The Vevo(R) 2100 System with MicroScan™ transducer, hVisualSonics Inc, Toronto, Canada) ultrasound in the contralateral (right) carotids. A month after ligation mice were sacrificed and both carotid arteries were dissected. The diameter of the carotids and other physical and biochemical variables were measured by myography. The animals were killed by an overdose of pentobarbiturate (Mebunat). Arteries were dissected out, used immediately for myography analyses, or either snap-frozen in liquid nitrogen or fixed in 4% paraformaldehyde. Histology images of cross-sectioned arteries were captured using a slide scanner (Pannoramic 250, 3DHISTECH Ltd) and cross-sectional sections were analysed using the CaseViewer 2.1 software (3DHISTECH). Measurements such as artery wall thickness (all tunicas), and VSMC thickness were also performed using the CaseViewer 2.1 software and plotted using PRISMS6. At least 20 random measurements were done per section in at least 3 individual samples per group. Measurements were performed in a blind fashion by 3 separate observers as the identity of each sample was a given number that only after measurements was used to identify to which group each sample belonged.

### Immunofluorescence of histological sections

Whole-mount fixed carotid sections were boiled in citric buffer (10 mM sodium citrate, 0.05% Tween-20, pH 6.0) for 10 min, washed and blocked with 10% normal goat serum (NGS) in PBS. Cells were permeabilized in the last wash with PBS-Triton (0.1%) and nuclei were stained with DAPI (Vector Laboratories, Burlingame, CA) (dilution 1:10,000) for 5 min before mounting with Vectashield antifading medium (Vector laboratories). Images were captured on a Pannoramic fluorescent scanner (Midi FL slide scanner, 3DHISTECH Ltd) and analysed using the CaseViewer 2.1 software (3DHISTECH).

### RT-PCR and genotyping

Standard protocols were followed. Briefly, RT-PCR amplification of total mRNA extracted from carotids and purified using TRIsure and phenol-chloroform was performed with AMV-reverse transcriptase (Promega) following the manufacturer’s instructions (see supplemental materials for primer information). Genotyping was performed using BioTools polymerase and buffer by screening the samples as previously described^14^.

### Gene expression

Quantitative real-time polymerase chain reaction (qPCR) was performed to analyse the gene expression levels Notch signalling markers (Jagged-1 receptor: JAG1, Notch-1 ligand: NOTCH1, Hey1 transcription factor: HEY1 and Hes1 transcription factor: HES1) and extracellular matrix proteins (fibronectin: FN, collagen type I: COL1 and collagen type III: COL3).

**Table:** 

GENE	LEFT PRIMER	RIGHT PRIMER	PROBE (TAQMAN)
Notch1	ctggaccccatggacatc	aggatgactgcacacattgc	#80, cat.no. 04689038001
Notch3	agctgggtcctgaggtgat	agacagagccggttgtcaat	#9, cat.no. 04685075001
Hes1	acaccggacaaaccaaagac	cgcctcttctccatgatagg	#99, cat.no. 04692179001
Hey2	gtggggagcgagaacaatta	gttgtcggtgaattggacct	#104, cat.no. 04692225001
Hey1	acgacatcgtcccaggttt	actgttattgattcggtctcgtc	#72, cat.no. 04688953001
Jag1	tggccgaggtcctacactt	gccttttcaattatgctatcagg	#22, cat.no. 04686969001
Dll4	aggtgccacttcggttacac	gggagagcaaatggctgata	#106, cat.no. 04692250001
Dll1	gggacagaggggagaagatg	tccatgttggtcatcacacc	#20, cat.no. 04686934001
ACTA2	taacccttcagcgttcagc	acatagctggagcagcgtct	#20, cat.no. 04686934001
Vimentin	ccaaccttttcttccctgaac	ttgagtgggtgtcaaccaga	#109, cat.no. 04692284001
Elastin	gctgctgctaaggctgctaa	agcacctgggagcctaactc	#67, cat.no. 04688660001
GADPH	gacaatgaatacggctacagca	ggcctctcttgctcagtgtc	#77, cat.no. 04689003001

### Computational approach for simulation of Notch signaling in arteries

Notch signaling in arteries was simulated by adopting the computational approach proposed in Loerakker *et al*. (PNAS, 2018), based on Sprinzak *et al*. (Nature, 2010) and Boareto *et al*. (PNAS, 2015). Briefly, due to symmetry, only a 1D series of communicating cells distributed through the arterial thickness was considered. The first cell, starting from the arterial luminal side, represented the endothelial layer, in contact with a number of interacting VSMCs. The levels of Notch and Delta present in the endothelial cell were not analysed, while its Jagged1 content was assumed to be constant and equal to a value *J*_*EC*_. Notch signaling occurring among the communicating VSMCs was modelled with a system of ordinary differential equations:

1 $$\{\begin{array}{ccc}\frac{d{N}_{i}}{dt} & = & {N}_{pr}{H}^{s}({I}_{i},{\lambda }_{N},{n}_{N})-{k}_{c}{N}_{i}{D}_{i}-{k}_{t,D}{N}_{i}{D}_{ext,i}-{k}_{c}{N}_{i}{J}_{i}-{k}_{t,J}{N}_{i}{J}_{ext,i}-\gamma {N}_{i},\\ \frac{d{J}_{i}}{dt} & = & {J}_{pr}{H}^{s}({I}_{i},{\lambda }_{J},{n}_{J})-{k}_{c}{J}_{i}{N}_{i}-{k}_{t,J}{J}_{i}{N}_{ext,J,i}-\gamma {J}_{i},\\ \frac{d{D}_{i}}{dt} & = & {D}_{pr}{H}^{s}({I}_{i},{\lambda }_{D},{n}_{D})-{k}_{c}{D}_{i}{N}_{i}-{k}_{t,D}{D}_{i}{N}_{ext,D,i}-\gamma {D}_{i},\\ \frac{d{I}_{i}}{dt} & = & {k}_{t,D}{N}_{i}{D}_{ext,i}+{k}_{t,J}{N}_{i}{J}_{ext,i}-{\gamma }_{I}{I}_{i}.\end{array}$$

This system of equations described the dynamics, over time *t*, of Notch (*N*), Jagged (*J*), Delta (*D*), and Notch Intracellular Domain (NICD, *J*) present in each VSMC, indexed with *i*. In Eq. 1, the parameter *k*_*c*_ is the cis-interaction rate of Notch with either Jagged or Delta; *k*_*t,J*_ and *k*_*t,D*_ describe the trans-interaction of Notch with Jagged and Delta, respectively; the parameters *N*_*pr*_, *J*_*pr*_, and *D*_*pr*_ represent the basal production rates of Notch, Jagged, and Delta, respectively; *γ* describes the degradation of these proteins; and *γ*_*I*_ corresponds to the degradation rate of NICD. The production rates of Notch, Jagged, and Delta were assumed to depend on the concentration of NICD, with this relationship described by the function

2 $${H}^{s}(I,\,\lambda ,n)=\lambda +\frac{1-\lambda }{1+{(I/{I}_{0})}^{n}},$$

where *λ*, *n*, and *I*_0_ are constant parameters. Furthermore, the basal production rates of Notch and Jagged were influenced by the strain experienced by VSMCs, such that

3 $$\{\begin{array}{ccc}{N}_{pr} & = & {N}_{pr,0}\,\exp ({A}_{N}\frac{{\varepsilon }_{p}}{{\sigma }_{p}}{\sigma }_{\theta }),\\ {J}_{pr} & = & {J}_{pr,0}\,\exp ({A}_{J}\frac{{\varepsilon }_{p}}{{\sigma }_{p}}{\sigma }_{\theta }).\end{array}$$

Here, *N*_*pr*,0_ and *J*_*pr*,0_ are the Notch and Jagged basal production rates in case of no strain experienced by cells, while *A*_*N*_ and *A*_*J*_ are parameters describing the decrease in production in response to strain. Additionally, *ε*_*p*_ and *σ*_*p*_ are parameters corresponding to the average physiological *in vivo* circumferential strain and stress of carotid arteries, respectively, and *σ*_*θ*_ is the average circumferential stress in the artery to simulate. This last value was computed with the Laplace’s law

4 $${\sigma }_{\theta }=\frac{pr}{h},$$

where *p*, *r*, and *h* are respectively the pressure, internal (lumen) radius, and wall thickness of the artery. The values of *p* and *r* were assumed to correspond to the average physiological *in vivo* values of these parameters, while the value of *h* was computed for each simulated artery as the number of VSMCs used in the simulation multiplied by 0.01 mm, corresponding to the assumed thickness of each VSMC layer, and then summed with the thickness of the endothelial layer (0.01 mm).

In Eq. 1, the values of *J*_*ext,i*_ and *D*_*ext,i*_ describe the amount of Jagged and Delta proteins that are present on the membranes of the cell neighbors of the *i*-th VSMC and that can interact with the Notch present in such VSMC. Similarly, the values of *N*_*ext,J,i*_ and *N*_*ext,D,i*_ represent the amount of Notch of neighboring VSMCs that can interact with Jagged and Delta proteins present on the membrane of the *i*-th VSMC. While Notch and Delta were assumed to be homogenously distributed on the cellular membrane, Jagged was assumed to cluster in each VSMC towards the outer arterial side. As a consequence of this assumption, the values of *J*_*ext,i*_, *D*_*ext,i*_, *N*_*ext,J,i*_, and *N*_*ext,D,i*_ depended on the amount of Notch, Delta, and Jagged in neighbouring VSMCs as follows

5 $$\{\begin{array}{ccc}{J}_{ext,i} & = & {J}_{i-1},\\ {D}_{ext,i} & = & \frac{1}{2}({D}_{i-1}+{D}_{i+1}),\\ {N}_{ext,J,i} & = & \frac{1}{2}{N}_{i+1},\\ {N}_{ext,D,i} & = & \frac{1}{2}({N}_{i-1}+{N}_{i+1}).\end{array}$$

The parameters chosen for the computational simulations (Table 1) corresponded to the parameters proposed in Loerakker *et al*. (2018) for young human carotid arteries, with parameter variations used to investigate the effects of vimKO, as described in the main text.

**Table 1:** Parameter values used to simulate human carotid arteries.

Parameter	Value	Parameter	Value	Parameter	Value
*N*_*pr,0*_	1400 h^−1^	*λ*_*N*_	2.0	*A*_*N*_	−5.79
*J*_*pr,0*_	1600 h^−1^	*λ*_*J*_	2.0	*ε*_*p*_	7.5%
*D*_*pr*_	100 h^−1^	*λ*_*D*_	0.0	*σ*_*p*_	107 mmHg
*k*_*c*_	5 × 10^−4^ h^−1^	*n*_*N*_	2.0	*r*	3.2 mm
*k*_*t,J*_	2.0 × 10^−5^ h^−1^	*n*_*J*_	5.0	*P*	16 kPa
*k*_*t,D*_	2.5 × 10^−5^ h^−1^	*n*_*D*_	2.0	*J*_*EC*_	4000
*γ*	0.1 h^−1^	*I*_0_	200		
*γ*_*I*_	0.5 h^−1^	*A*_*J*_	−4.17		

As in Loerakker *et al*. (PNAS, 2018) and Boareto *et al*. (PNAS, 2015), random values between 0 and 6000 molecules were chosen for *N*, *J*, and *D* as initial conditions. Similarly, random values between 0 and 600 molecules were chosen as initial condition for *I*. The system of ordinary differential equations was then solved for 0 < *t* ≤ 250 h, with an explicit time integration scheme and a time step equal to 0.01 h. The simulations were performed for arterial walls comprising 1 to 100 VSMC layers. For each number of layers, the simulations were repeated 25 times, each time randomly choosing the initial conditions. Finally, for each number of layers, the values of *N*, *J*, *D*, and *I* were averaged across the results of these 25 simulations.

### Statistical analyses

Charts and statistical analyses were performed using PRISM6 (Graphpad software). ANOVA was used for statistical analyses when comparing several groups, followed by Tukey-Kramer multiple comparisons post hoc test to identify the significantly different groups. When comparing two groups, statistical significance was determined using Student’s t-test. Error bars present SD or SEM as indicated by figure texts and p < 0.05 was considered statistically significant.

## Supplementary information

Supplementary file
