# Supplementary material for: Vimentin regulates Notch signaling strength and arterial remodeling in response to hemodynamic stress
Source: Sci Rep. 2019 Aug 27;9:12415. doi: 10.1038/s41598-019-48218-w (PMC6712036; doi:10.1038/s41598-019-48218-w)
Supplement: Supplementary file 1 — Supplementary file [file 41598_2019_48218_MOESM1_ESM.docx]

**Vimentin regulates Notch signaling strength and arterial remodeling in response to hemodynamic stress**

Nicole C.A. van Engeland^1,2,#^, Freddy Suarez Rodriguez^1,3, #^, Adolfo Rivero-Müller^1,4^, Tommaso Ristori^1,2,7^, Camille L. Duran^5^, Oscar M.J.A. Stassen^1,3^, Daniel Antfolk^1,3^, Rob Driessen^2^, Saku Ruohonen^6^, Suvi T. Ruohonen^6,8^, Salla Nuutinen^6^, Eriika Savontaus^6,8^, Sandra Loerakker^2,7^, Kayla J. Bayless^5^, Marika Sjöqvist^1,3^, Carlijn V.C. Bouten^2,7^, John E. Eriksson^3^, Cecilia M. Sahlgren*^1,2,3,7^

^1^ Åbo Akademi University, Faculty of Science and Engineering, Biosciences, Turku, Finland, ^2^ Eindhoven University of Technology, Department of Biomedical Engineering, 5600 MB Eindhoven, the Netherlands, ^3^ Turku Bioscience, Åbo Akademi University and University of Turku, Finland, ^4^ Department of Biochemistry and Molecular Biology, Medical University of Lublin, Poland, ^5^ Department of Molecular & Cellular Medicine, Texas A&M University Health Science Center, College Station, TX, 77843, USA, ^6^ Institute of Biomedicine, Research Centre for Integrative Physiology and Pharmacology, University of Turku, Finland. ^7^ Institute of Complex Molecular Systems, Eindhoven University of Technology, Eindhoven, The Netherlands, ^8^ Turku Center for Disease Modelling, University of Turku, Finland.

# Equal contribution

*Correspondence:

Cecilia M. Sahlgren

Tel no: +358 407713952

E-mail: [cecilia.sahlgren@abo.fi](mailto:cecilia.sahlgren@abo.fi), c.m.sahlgren@tue.nl

**Supplementary materials and methods**

**Time point shear stress experiments.**

HUVEC cells were seeded into three wells of seven 6-well plates with 2mL of endothelial cell media 2 (Promocell). After confluence, the media of every plate was change. Three of the plates were taken into an orbital well shaker system at 100rpm, three were kept as static controls, and the last one was washed with PBS, lysated on 2xLSB and store as the control for 0 hours. Then at 6, 12 and 24 hours one plate of static and flow was also washed in PBS, lysated and store. All the samples were boiled 10min at +95°C and analyzed by weseter blotting using Vimentin V9 (Santa Cruz sc-6260), Vimentin Phospho-Vimentin (ser39) (#13614 CST), Vimentin (phoshpho S55 ab22651) and HSC-70 (Enzo N27F3-4).

**Notch transactivation in vimentin phosphomutants.**

Jagged1 overexpressing HEK293 cells (293JAG) were plated at 120 000 cells per well in a 12-well plate. 1.5x106 full-length Notch overexpressing cells (293FLN) were plate in a 10cm culture dish. After 24 hours the 293JAG cells were transfected with different Vimentin phosphomutant plasmids and the 29FLN with 12CSL-luciferase using JetPRIME©. After 24 hours the 293FLN were seeded directly on top and co-cultured for 24 hours. Afterwards the cells were lysed and luciferase reporter activity was measured with a Biotek Synergy plate reader using a luciferase Assay (Promega).

**Immunoprecipitation**

HUVEC were seeded in 6-well plates and upon confluence subjected to shear stress with an orbital shaker at 100 rpm (Filipovic, CMBBE 2016). After exposure to shear stress HUVEC cells were harvested in lysis buffer (described in Lähdenniemi et al 2017 (63)) and incubated on ice for 30 min. After preclearing, the lysate was incubated with Jagged1 antibody (28H8 rabbit, Cell Signaling) or IgG control. Protein G sepharose beads were added to the samples and incubated at +4°C overnight. Samples were washed with TEG buffer (20 mM Tris-HCl (pH 7.5), 1 mM EDTA, 10% Glycerol) and resuspended in Laemmli sample buffer for analysis by SDS-page and western blotting. Vimentin (V9, Sigma-Aldrich), Jagged1 (28H8, Cell Signaling), Vimentin V9 (Santa Cruz sc-6260), Vimentin Phospho-Vimentin (ser39) (#13614 CST), and β-actin (4967L, Cell Signaling) were used for immunoblotting.

**Uniaxial strain**

Cyclic strain was applied to VSMCs by using a FX-5000 Flexcell system (Flexcell International Corporation). First, uniflex plates were coated with 5 μg/cm^2^ fibronectin (FN) in phosphate buffered saline (PBS) for 30 minutes at 37 °C and then washed two times with PBS. After washing, wells were covered with 1% pluronic (Sigma) for 30 minutes at room temperature and washed with PBS twice. Cells were seeded at 20,000 cells/well and incubated overnight at 37°C to let them attach to the membrane. To apply uniaxial cyclic strain, a vacuum was applied and the membranes were stretched over rectangular loading posts oriented in the y-direction (90°). Strains of 10% (1 Hz, sine wave) were applied in the x direction (0°) for 48 hours. Unstrained samples, cultured under the same conditions and on identical uniflex plates, were used as control group. Samples were analysed for protein- and gene expression.

**Proximity ligation assay**

Cells were fixed for 10 min with -20°C methanol and 1 min with acetone before incubating with vimentin (Vimentin V9, Abcam) and Jagged1 (Jagged1 28H8, Cell Signaling) primary antibodies for 75 min at RT (3 µL antibody per 100 µL blocking solution). Thereafter, the assays were continued using Duolink DUO92102 reagents (Sigma-Aldrich) according to manufacturer’s instructions. Finally, samples were mounted with DAPI-containing medium (Duolink DUO82040, Sigma-Aldrich) and imaged using a Carl Zeiss LSM780 confocal microscope.

**Antibodies**

Antibodies used for western blotting: vimentin (D21H3) (rabbit, Cell signaling), jagged1 (28H8) (rabbit, Cell signaling), Notch3 (rabbit, Cell signaling) and beta-actin (rabbit, Cell signaling).

**Myography**

Rapidly after euthanasia, the carotid arteries of VimKO and WT mice were placed in ice-cold oxygenated Krebs solution. Segments (2 mm in length) of carotid arteries were studied by wire myography (Danish Myograph Technologies, Aarhus, Denmark) for arterial diameter, and contractile responses to phenylephrine (PE), and endothelium-dependent and -independent relaxations to acetylcholine (ACh) and sodium nitroprusside (SNP), respectively, as previously described(64). After mounting, vessel segments were allowed to equilibrate for at least 20 minutes before normalisation to 100 mmHg physiological tension. During the functional measurements, the distance between the two wires was kept constant and the responsiveness (change in vessel diameter by contraction and relaxation) of the vessels to different stimuli was studied. Isolated arteries were contracted three times with 62 mM KCl to determine the maximal contraction of the vessels. The vasoconstrictive response was determined by cumulative doses of phenylephrine. Vessels were stimulated with 1 mM prostaglandin F_2α_ to obtain 50-80% of the maximal reference contraction to KCl to quantify acetylcholine-induced endothelium-dependent vasodilatation. Endothelium-independent relaxation was studied in the presence of sodium nitroprusside (SNP), which acts as a vasodilator by releasing NO. The contribution of NO on vasoconstriction and vasodilatation was determined by incubating vessels with N^ω^-Nitro-L-arginine (L-NNA, 100 µM) 30 minutes before contracting the vessels with phenylephrine, and subsequently relaxing them with ACh. Between the functional experiments, the vessel baths were washed at least three times with fresh, warm, and oxygenated Krebs solution (119 mM NaCl, 25 mM NaHCO_3_, 5.5 mM glucose, 4.7 mM KCl, 1.2 mM KH_2_PO_4_, 1.2 mM MgSO_4_ ∙ 7H_2_O, and 2.5 mM CaCl_2_ ∙ 2H_2_O).

After functional experiments, vessel segments were equilibrated 30 minutes and studied for mechanical properties by a graded passive distension procedure. The two wires were brought together so that no tension was placed in the ring preparations and the micrometer was zeroed (L_0_). Then, the distance between the wires was increased stepwise by 10 µm every 60 seconds until 30-40 mN wall force was reached. The wall force and micrometer reading were recorded at each step, and the related wall stress (mN/mm) was calculated and plotted against the resulting strain (ΔL/L_0_)(65–67).

During the functional and mechanical experiments, vessel segments were kept in aerated Krebs solution (95% O_2_ and 5% CO_2_) and warmed to 37°C. Krebs solution in the myograph wells was discarded at least every 20 minutes and fresh, warm, and oxygenated Krebs solution was provided to maintain the physiological properties of the vessels. Chart5 and PowerLab (ADI Instruments, Colorado Springs, CO) were used for data recording and analysis.

**Supplemental figures titles and legends**

**
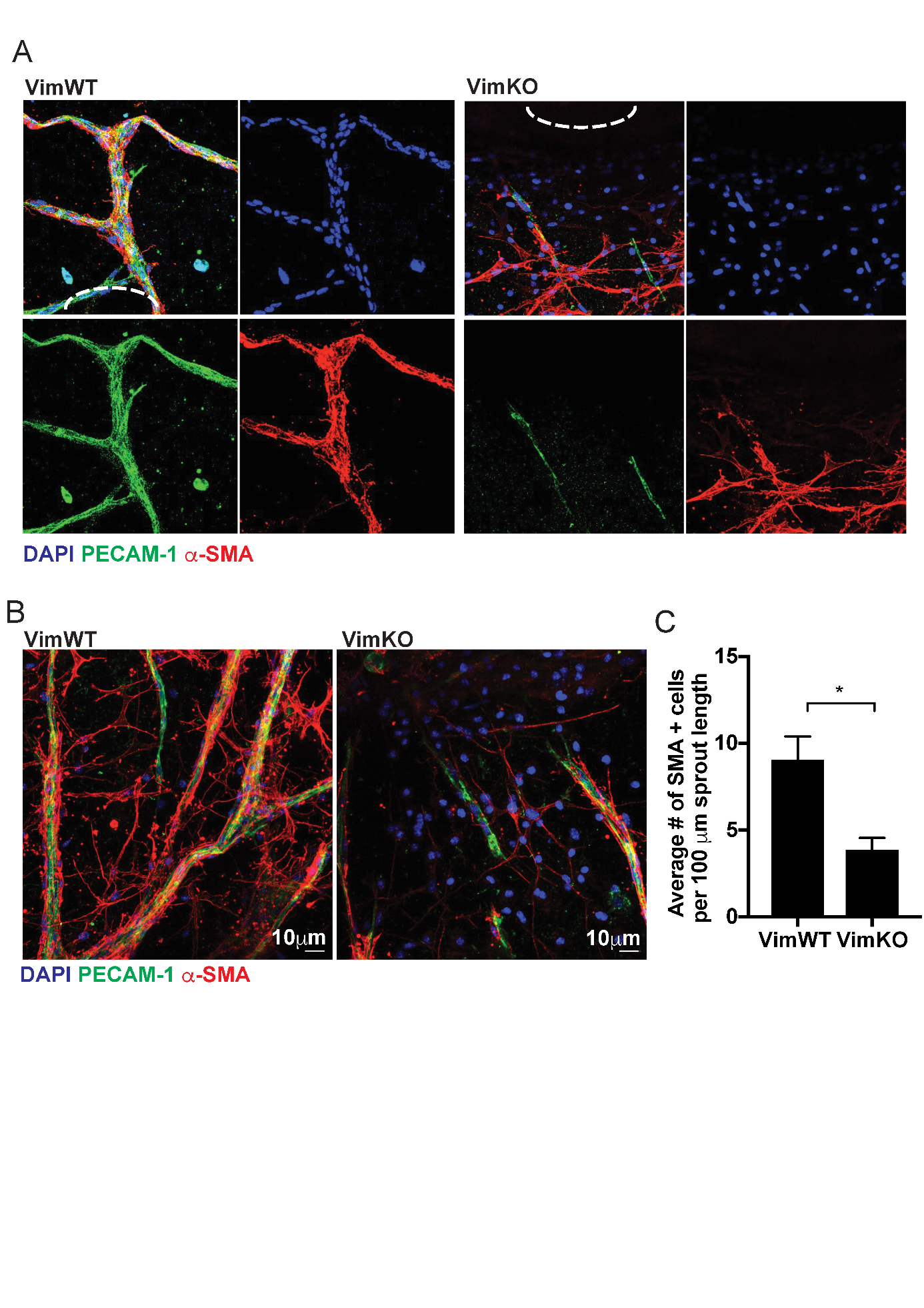
Supplementary figure 1. Vimentin is important for VSMC coverage**

Aortic ring assays were performed using aortae from VimWT and VimKO mice. After 6 days, rings were fixed, permeabilized, and stained with DAPI (blue) and antibodies directed to PECAM-1 (green) and alpha-smooth muscle actin (αSMA, red). **(A,B)** Using confocal microscopy, Z-stack images were captured with a 1 µm step size. Representative images as max projections are shown. Dotted white line represents edge of aortic ring where growth initiates. Scale bar represents 10 µm. **(C)** Using Z-stacked images captured in (A and B), the number of αSMA positive cells along the length of the PECAM-1 positive structure was quantified. Data represent the average number of αSMA-positive cells per 100 µm EC sprout length. Error bars represent SEM. Statistical significance was determined using Student’s t-test, p<0.05

**
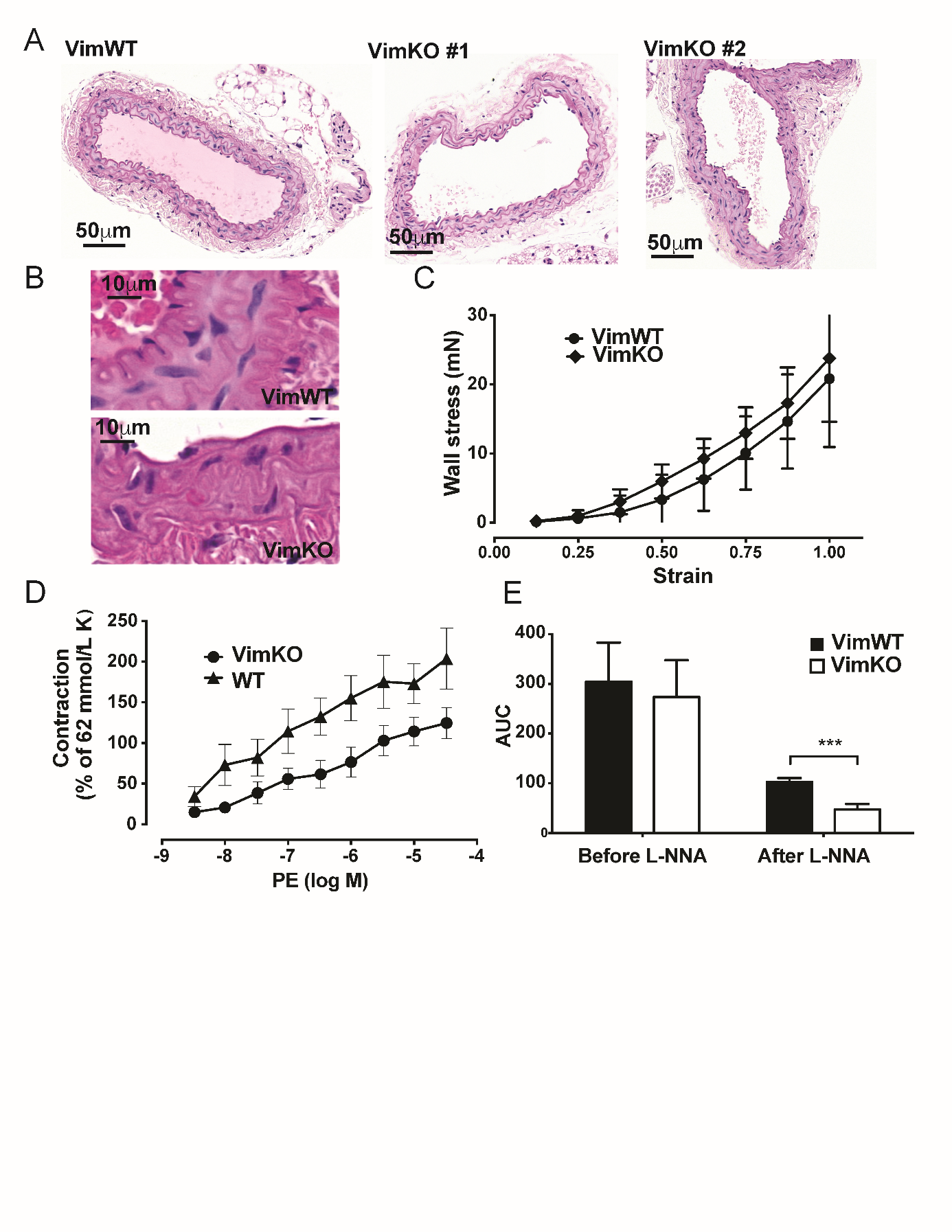
Supplementary figure 2. Vimentin knockout arteries are disorganized, stiffer and show disrupted responses to contractile and relaxation stimuli. (A)** Haematoxylin and eosin (H&E) staining of histological sections of carotid arteries from VimWT and VimKO mice. The architecture of the vascular wall in VimKO is disorganized as compared to the clear lamellar organisation of VSMC in VimWT. **(B)** Higher magnification images of H&E stained arteries from VimWT and VimKO mice. **(C)** The stiffness of VimWT and VimKO arteries was analyzed by wire myography of isolated carotid arteries. The VimKO arteries were stiffer although the difference was not statistically significant. Error bars represent SD, mN is milliNewton. **(D-E)** Vasoconstriction and vasodilatation was determined by incubating VimWT and VimKO vessels with N^ω^-Nitro-L-arginine (L-NNA, 100 µM) before contracting the vessels with phenylephrine, and subsequently relaxing them with ACh. The VimWT arteries showed increased contractile responses (D), whereas endothelium dependent relaxation of VimKO is impaired (E). Error bars represent SD, except in E where they are SEM. In all cases, the number of animals was n=4 per group. Statistical significance was analyzed by Student t-test. *** = p<0.001, * = p<0.05).

**
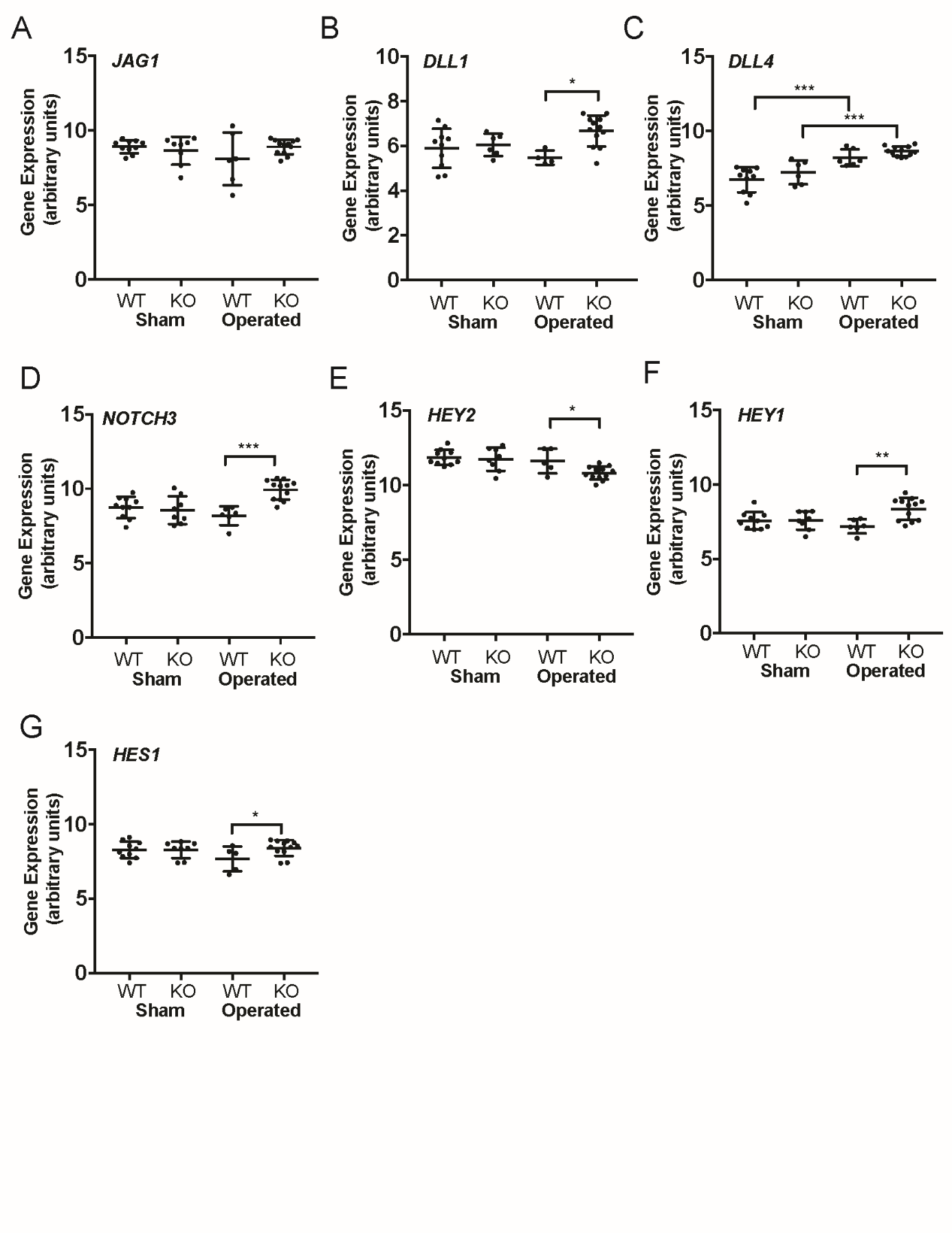
Supplementary figure 3. VimKO mice display disrupted Notch signaling profiles in remodeling arteries. (A-G)** Q-PCR analyses of *Jagged1*, *Dll1*, *Dll4*, *Notch3*, *Hey2, Hey1*, and *Hes 1,* expression in isolated VimWT and VimKO carotid arteries 4 weeks after carotid ligation. *Hey2* was reduced in response to ligation in the contralateral VimKO artery. Expression of *Jagged1* was not changed in response to ligation or vimentin depletion. On the contrary, expression of *Dll1*, *Dll4*, *Notch3*, *Hes1*, and *Hey1* was increased in the VimKO contralateral artery after ligation. ANOVA was used for statistical analyses, followed by Tukey-Kramer multiple comparisons post hoc test to identify the groups differing. Data is presented as the mean ± SD, and p < 0.05 was considered statistically significant.

**
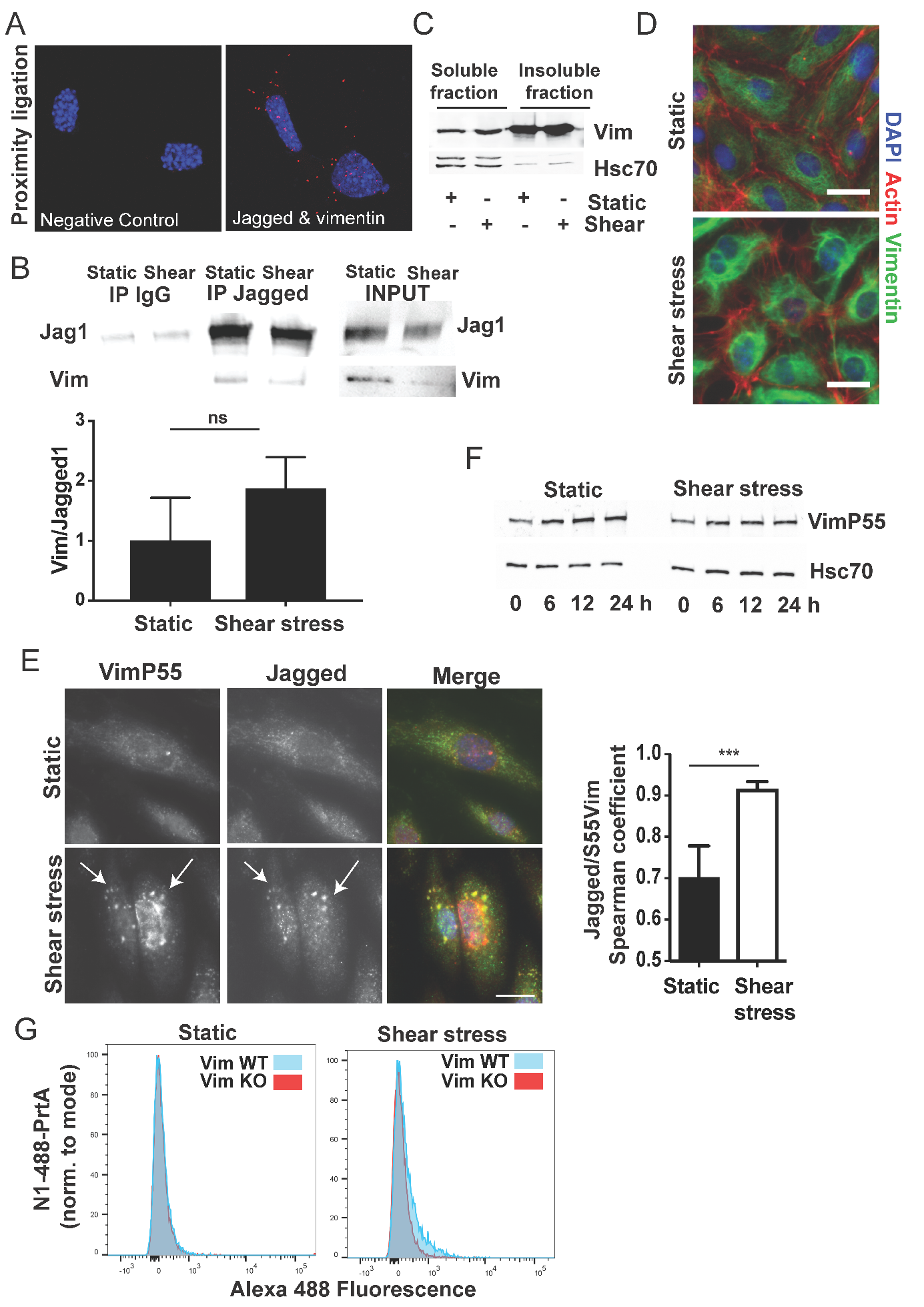
Supplementary figure 4. Regulation of Jagged1 and vimentin during shear stress**. **(A)** Representative image of a proximity ligation assay of vimentin and Jagged1 in ECs. Red dots indicate proximity between the proteins (left image). The left image is the negative control. **(B)** Immunoprecipitation of Jagged1 in ECs cultured under static conditions or exposed to shear stress. Immunoblotting was performed using an antibody recognizing vimentin. **(C)** Shear stress does not affect vimentin polymerization. Western blot demonstrates vimentin levels in the soluble and insoluble fractions after detergent extraction of ECs cultured under static and shear stress conditions. Full western blots in supplementary figure 8. **(D)** Vimentin organization in ECs exposed to shear stress or cultured under static conditions. ECs were exposed to shear either in an IBIDI shear system or in an orbital shaker. Upon shear stress vimentin distribution in the cell changed. The vimentin network retracted from the cell edges (indicated by vimentin negative actin positive cell borders) under shear stress. Scale bars are 50 μm **(E)** ECs were exposed to shear stress and compared with static controls for vimentin and Jagged1 localization. Shear stress induced clustering of Jagged1 as previously described in (60). Scale bars are 50 µm. Jagged1 clusters co-localize with vimentin subunits phosphorylated at serine 55. Phosphorylated vimentin was detected in ECs exposed to shear stress by a phosphospecific vimentin antibody (Phospho Vimentin Ser56). Jagged1 co-localization with phosphorylated vimentin in static and shear stressed ECs was analyzed with the spearman colocalization coefficient. **(F)** Expression levels of vimentin phosphorylated at serine 55 in ECs exposed to shear stress as analyzed by western blotting using phosphospecific antibodies. **(G)** Representative histograms of three separate experiments of N1ECD-Jagged1 endocytosis in VimWT and VimKO cells during shear stress, as analyzed by FACS. Fluorescently labelled N1ECD was coupled to Protein A (PrtA) beads (N1-488-PrtA) in order to mimic the mechanical strain produced during receptor-ligand endocytosis and transactivation.

**
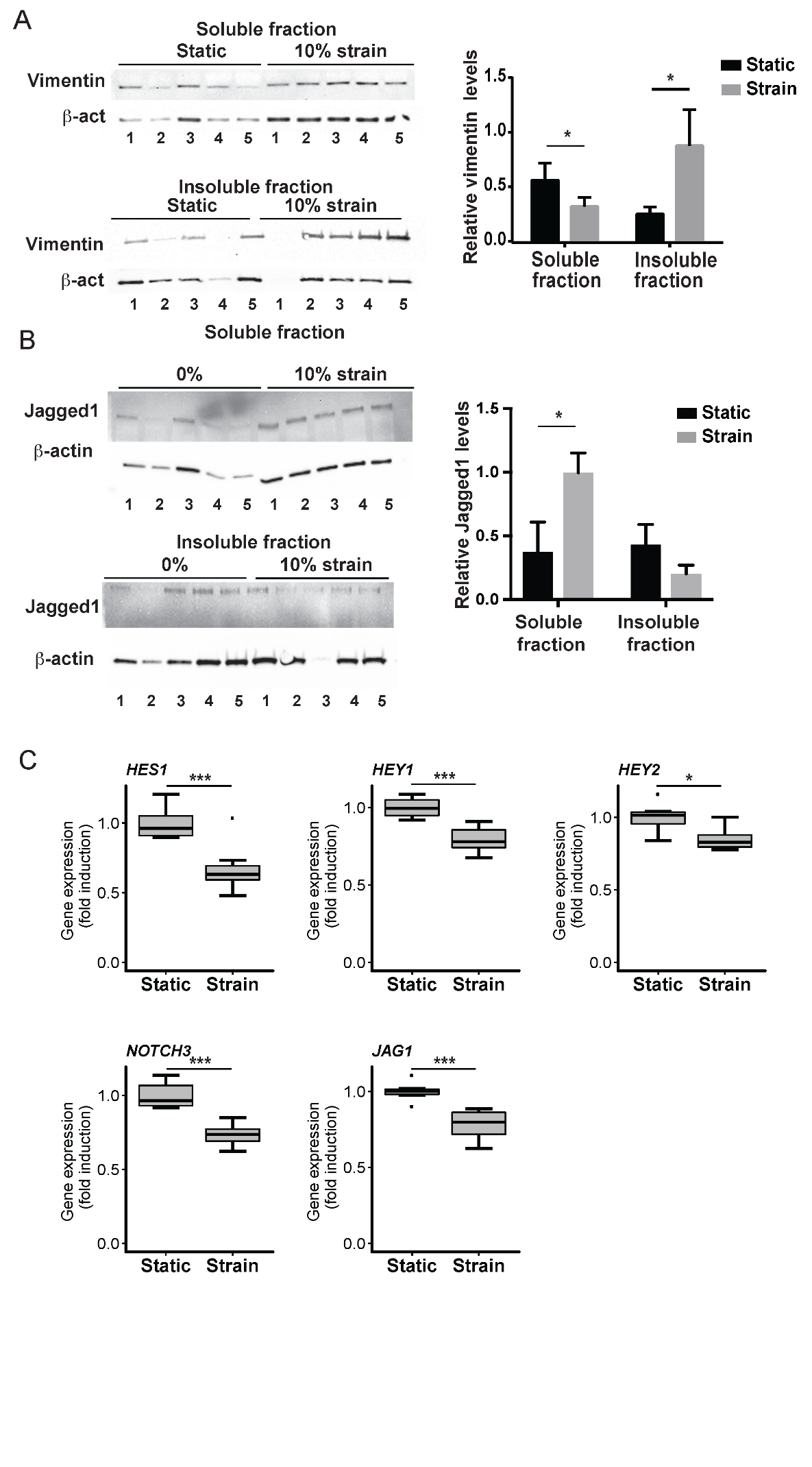
Supplementary figure 5. Strain induces vimentin polymerization and reduces Jagged1 expression and Notch signaling. (A-B)** Western blot and quantification of protein levels in the soluble and insoluble fraction in VSMCs exposed to uniaxial strain (10%) or static conditions (0%). **(C)** mRNA levels of Notch genes in VSMCs exposed to uniaxial strain (10%) or static conditions (0%). Statistical significance was analyzed by Student t-test. *** = p<0.001, ** = p<0.01, and * = p<0.05.

**

Supplementary figure 6. Jagged1 regulates arterial wall thickness.** Average NICD content for cells populating the wall of arteries of different thickness, as predicted by the computational simulation of Notch signaling with variations of the parameter associated with the Jagged1 production. The original model parameters are reported in the supplementary material. Upregulated Jagged1 production has little effects on the average NICD content and, consequently, on the arterial wall thickness, while decreasing Jagged1 production causes a large decrease in the NICD levels.

**
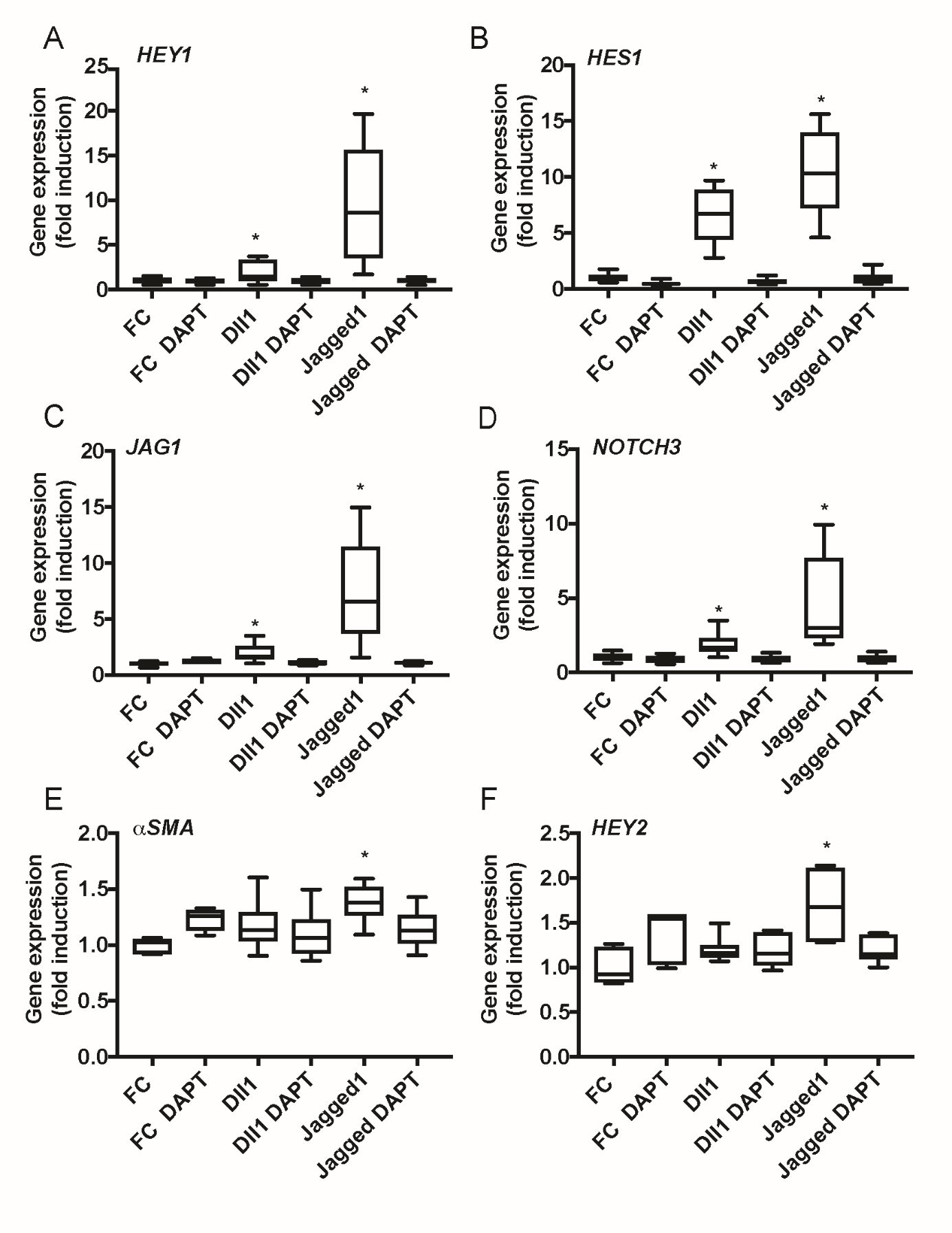
Supplementary figure 7. Expression of Notch target genes in VSMC activated by recombinant Dll1 or Jagged1.** VSMCs activated by immobilized recombinant Dll1 and Jagged1 ligands at a concentration of 10 μg/ml for 24 h in the presence and absence of the Notch inhibitor DAPT **(A-F)**. Expression of *Hey1* (A), *Hes1* (B), *Jagged1* (C), *Notch3* (D), *αSMA* (E), and *Hey2* (F) as analyzed by Q-PCR. Boxplots are presented as fold induction compared to expression in VSMCs cultured on the IgG-FC control, n=4. *p<0.05.

**
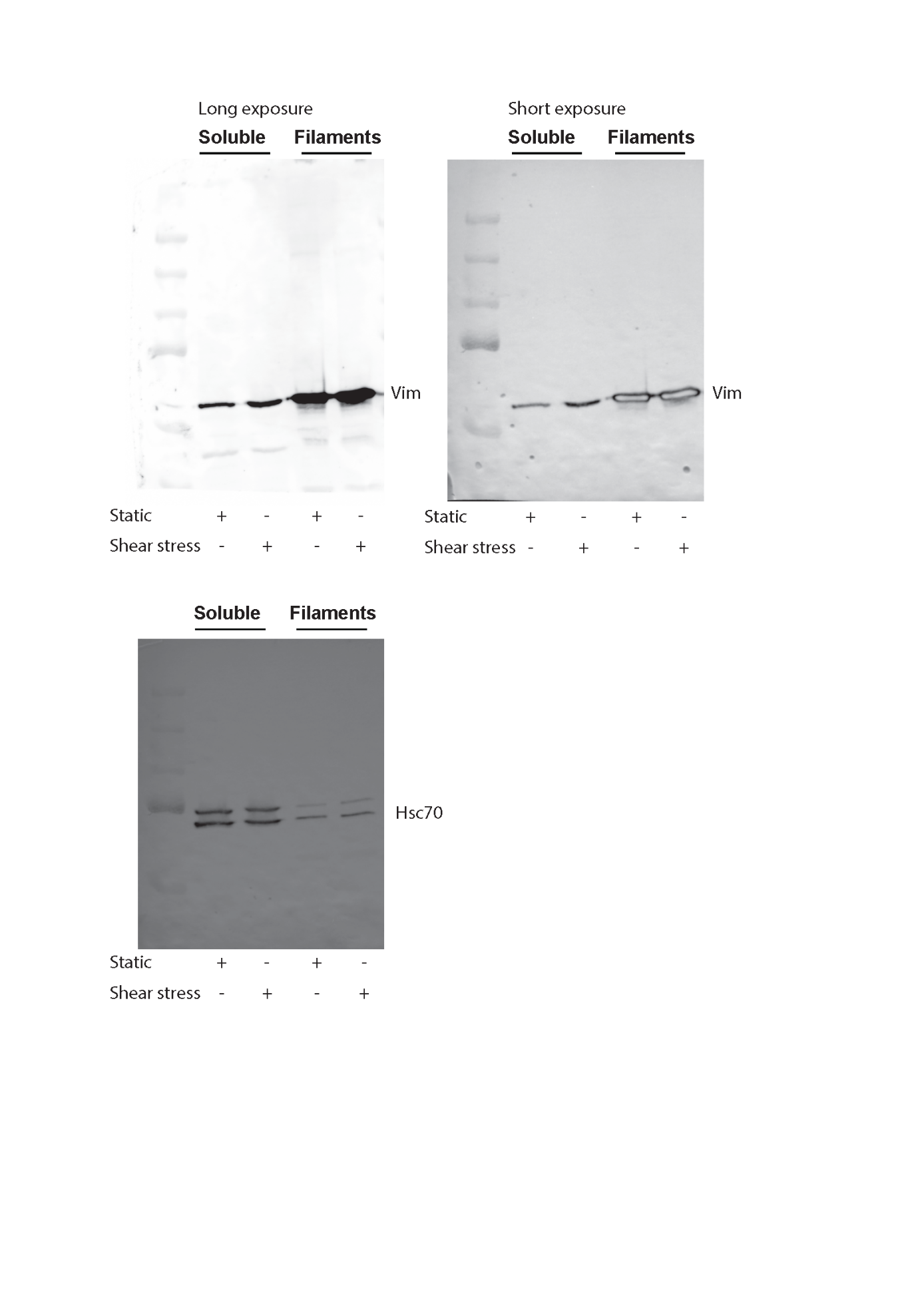
**

**Supplementary figure 8. Full western blot of vimentin and Hsc70.** Shear stress does not affect vimentin polymerization. Western blot demonstrates vimentin and Hsc70 levels in the soluble and insoluble fractions after detergent extraction of ECs cultured under static and shear stress conditions for different exposure times.

**
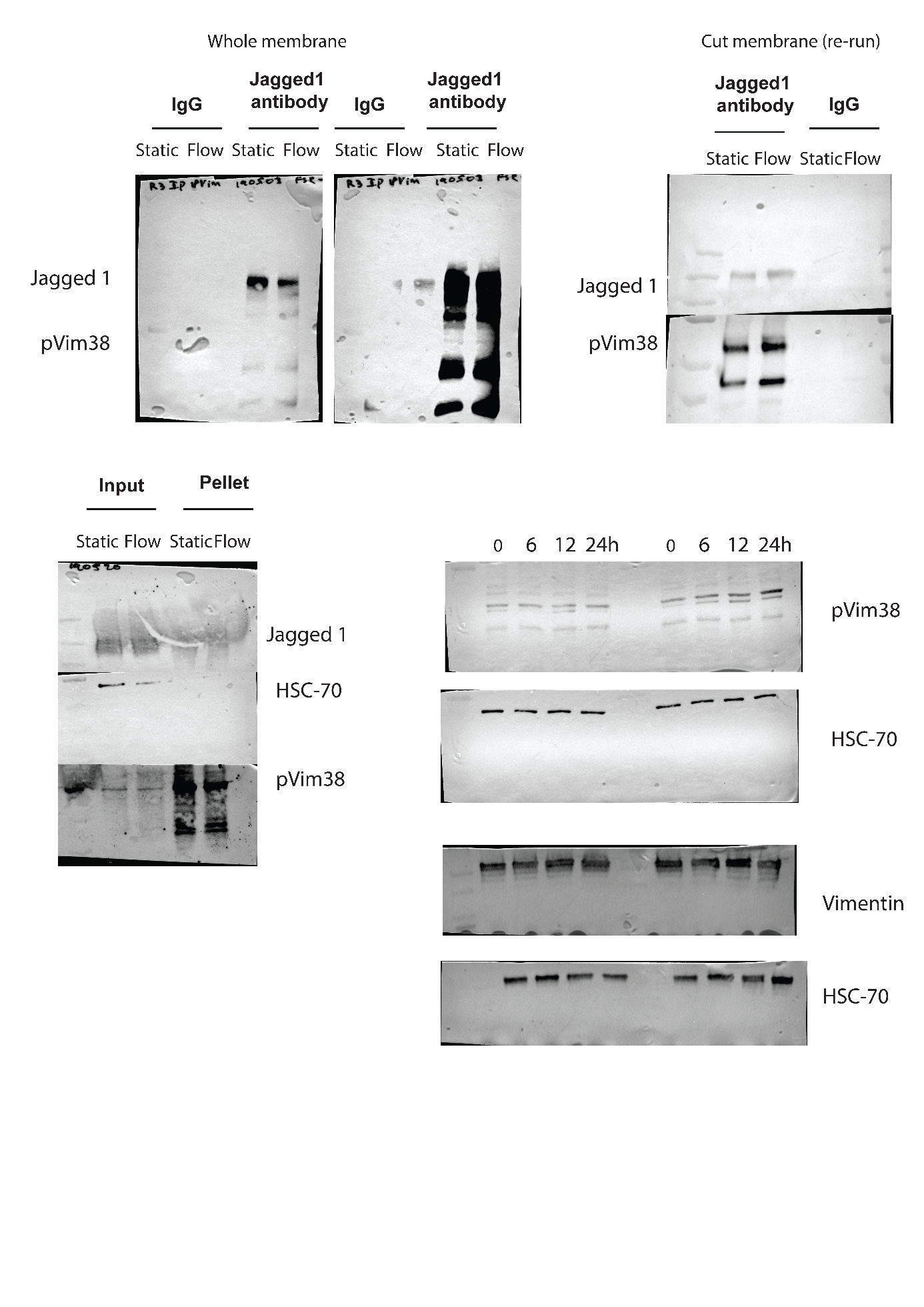
Supplementary figure 9. Full western blot of pVimS38, Jagged1 and HSC-70 for co-IP and timepoint experiments.** Phosphorylation of vimentin at serine 38 increase overtime when exposed to shear stress. Jagged1 interacts with pVimS38 during shear stress.
